# Observation of α-Synuclein Preformed Fibrils Interacting with SH-SY5Y Neuroblastoma Cell Membranes Using Scanning Ion Conductance Microscopy

**DOI:** 10.1021/acschemneuro.2c00478

**Published:** 2022-12-01

**Authors:** Christina Feng, Marisol Flores, Christina Dhoj, Adaly Garcia, Sheehan Belleca, Dana Abou Abbas, Jacob Parres-Gold, Aimee Anguiano, Edith Porter, Yixian Wang

**Affiliations:** † Department of Chemistry and Biochemistry, California State University, Los Angeles, Los Angeles, California 90032, United States; ‡ Department of Biological Sciences, California State University, Los Angeles, Los Angeles, California 90032, United States

**Keywords:** Parkinson’s disease, SICM, α-synuclein, protein−lipid interaction, XTT, LDH

## Abstract

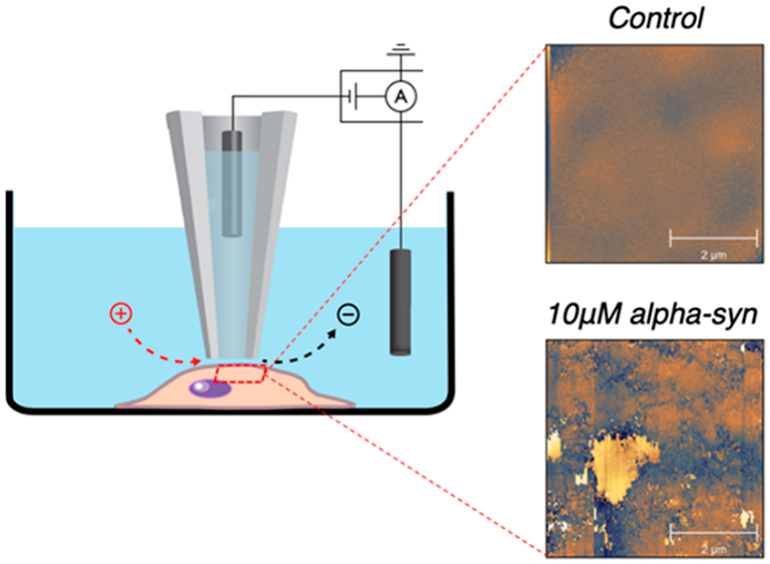

Parkinson’s disease (PD) is the second-most prevalent neurodegenerative disorder in the U.S. α-Synuclein (α-Syn) preformed fibrils (PFFs) have been shown to propagate PD pathology in neuronal populations. However, little work has directly characterized the morphological changes on membranes associated with α-Syn PFFs at a cellular level. Scanning ion conductance microscopy (SICM) is a noninvasive *in situ* cell imaging technique and therefore uniquely advantageous to investigate PFF-induced membrane changes in neuroblastoma cells. The present work used SICM to monitor cytoplasmic membrane changes of SH-SY5Y neuroblastoma cells after incubation with varying concentrations of α-Syn PFFs. Cell membrane roughness significantly increased as the concentration of α-Syn PFFs increased. Noticeable protrusions that assumed a more crystalline appearance at higher α-Syn PFF concentrations were also observed. Cell viability was only slightly reduced, though statistically significantly, to about 80% but independent of the dose. These observations indicate that within the 48 h treatment period, PFFs continue to accumulate on the cell membranes, leading to membrane roughness increase without causing prominent cell death. Since PFFs did not induce major cell death, these data suggest that early interventions targeting fibrils before further aggregation may prevent the progression of neuron loss in Parkinson’s disease.

Parkinson’s disease (PD) is the second-most prevalent neurodegenerative disorder in the U.S., behind Alzheimer’s disease.^1^ In PD, misfolded aggregates of the natively unstructured protein α-synuclein (α-Syn) are known to induce the death of dopamine-producing neurons in the substantia nigra, ultimately hindering motor function.^2^ α-Syn fibrils, an α-Syn aggregate associated with Lewy bodies in PD and typically 50–200 nm in length, have been shown to play an essential role in the prion-like spread of PD throughout neuronal networks and can cause cell dyshomeostasis over long periods.^3−6^ α-Syn fibrils have been shown to cause endogenous α-Syn to aggregate and accumulate, especially when neuronal cells originally express high levels of α-Syn.^7^

α-Syn preformed fibrils (PFFs) are α-Syn fibrils that are made by incubating and aggregating recombinant monomeric α-Syn under controlled conditions and are used to model α-Syn pathology.^8^ PFFs have been demonstrated to promote cellular uptake, seeding, and propagation of α-Syn both *in vivo* and *in vitro*.^9−11^ For example, previous work has shown that incubating α-Syn PFFs with neuronal cells promotes the intracellular aggregation of endogenous α-Syn at much higher levels than other aggregates.^3,9^ Additionally, PFFs seed aggregation in a time and dose-dependent manner, suggesting that longer incubation periods and higher fibril concentrations promote intracellular aggregation, which also correlates with decreased synaptic activity and eventual cell death.^9,12,13^ These findings demonstrate the intracellular changes associated with fibril treatment. On the other hand, several studies have investigated the direct interaction between PFFs and lipid membranes. Solid-state NMR has been used to study the binding affinity between fibrils and lipids, which has revealed that vesicles coaggregate with fibrils, especially when membranes contain anionic lipids.^14,15^ Related conclusions have been shown with cryoelectron microscopy (cryo-EM), atomic force microscopy (AFM), and fluorescence microscopy, which have characterized the coaggregation between fibrils and artificial lipids.^14,16−18^ However, these previous studies are primarily based on artificial lipid systems, and evidence from cell models is still lacking.

Scanning ion conductance microscopy (SICM) is a noninvasive imaging technique used to characterize the morphological features of cells in a liquid environment without damaging the cell membrane (Figure 1A).^19−21^ This technique utilizes a nanopipette (Figure S1) for probing. The probe–sample distance-dependent electric current decreases significantly when the pipet approaches very close to a surface. Typically, 99% of the baseline current is selected as the set point to maintain a constant distance between the pipet and sample surface during imaging (Figure 1A). As a result, the heights at various points on a surface can be measured and used to generate a three-dimensional topography. SICM is advantageous with its nanometer precision, chemical probes-free measurement, and negligible force on the sample and has been applied extensively for monitoring membrane structural changes of biological samples.^22−24^ We have previously demonstrated that SICM is capable of monitoring cellular membrane changes induced by α-Syn oligomers at SH-SY5Y cells.^25^

**Figure 1:**
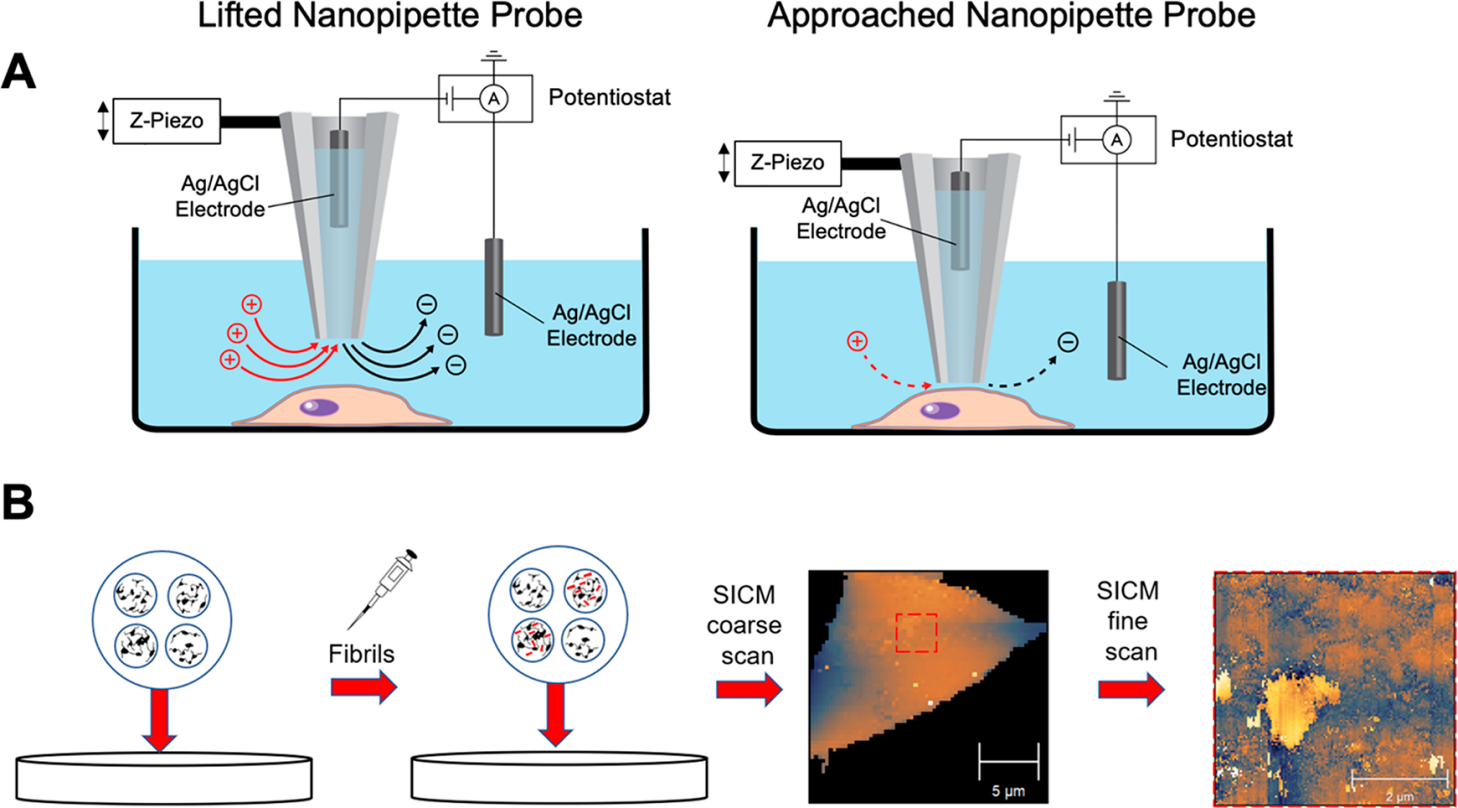
(A) Schematic of SICM. (B) Flowchart illustrating the SICM experiment procedure. PFFs (red) are added to wells containing SH-SY5Y cells and incubated for 48 h. SICM was then used to image cells, starting with a coarse whole-cell scan (64 × 64 pixels) and followed by multiple fine scans (128 × 128 pixels).

Here, we aimed to characterize interactions between synthetic α-Syn PFFs and SH-SY5Y neuroblastoma cell membranes using SICM and provide new insight into the mechanism of PFF-induced neuronal dysfunction. In contrast to work done with α-Syn oligomers, we exposed live cells to α-Syn PFFs for an extended time (48 h versus 2 h), as PFFs are known to be slower-acting compared to oligomers.^25−27^ Additionally, instead of imaging a single cell over time, we sampled multiple cells to expand our observations to cell populations. A schematic of the experimental setup can be found in Figure 1B. SH-SY5Y neuroblastoma cell membranes were treated with varying concentrations of α-Syn PFFs for 48 h and then imaged with SICM, followed by quantitative analysis of the subsequent morphological changes of neuronal membranes. In addition, cell viability was assessed with an XTT reduction assay and lactate dehydrogenase (LDH) release assay to assist in interpreting the SICM results.

The α-Syn PFFs used in this study were purchased, and their length distribution of the PFFs was characterized using AFM after dissolving them in cell culture media. Figure 2A and Figure 2B show the representative AFM images (2 μm × 2 μm) of the PFFs adhered to a mica substrate, and Figure 2C shows the histogram of the length of PFFs (*n* = 3290) acquired from 11 AFM images. Fifty-one percent of the PFFs are in the length range from 30 to 60 nm, and the mean ± 95% confidence interval is 74.7 ± 2.7 nm. They are comparable in size to the PFFs used in previous work from Cascella et al. (∼50 nm).^3^Figure S2 shows the vendor-provided circular dichroism (CD) spectra, which indicate significant β-sheet content in the PFFs (43.0%).

**Figure 2:**
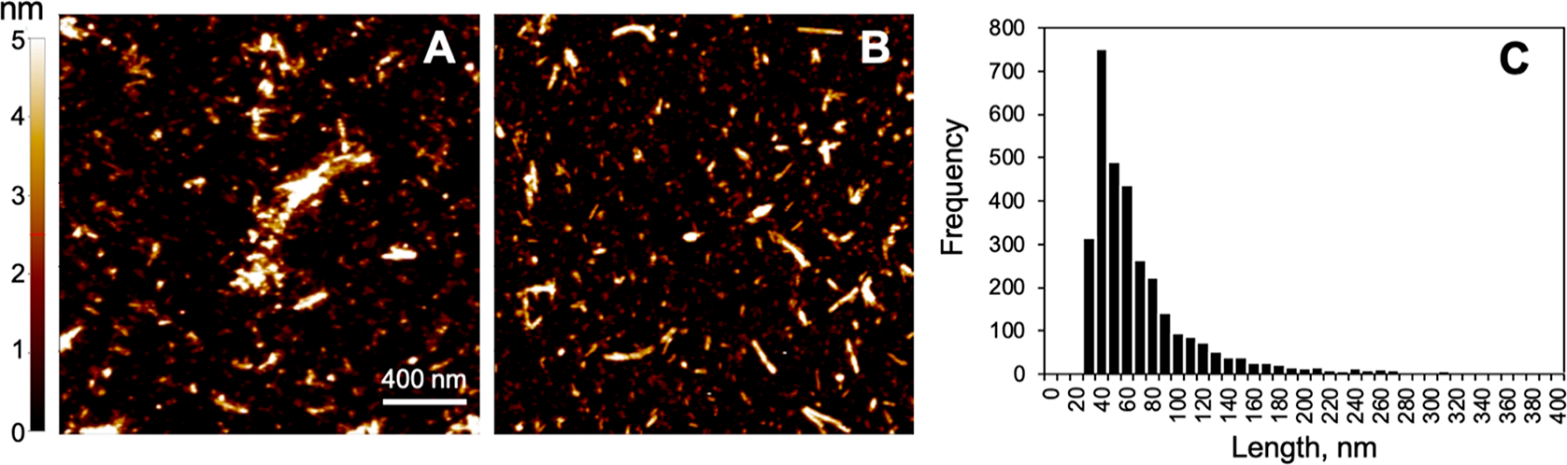
AFM characterization of PFFs. (A, B) Representative AFM images (2 μm × 2 μm) of α- Syn PFFs adhered to a mica substrate. Images were first-order flattened using XEI. (C) Histogram of the lengths of PFFs imaged with AFM (*n* = 3290).

Previous literature has indicated that exposure to 0.1–10 μM PFFs significantly decreases synaptic activity and increases reactive oxygen species generation.^3,9,28^ Therefore, this study exposed live SH-SY5Y cells to 1 μM, 5 μM, and 10 μM α-Syn PFFs added extracellularly to culture media for 48 h. Cells were then fixed in 1% paraformaldehyde for 30 min to preserve cell morphology and enable the scanning of multiple samples for 3–4 h. A total of 73 cell sections (5 μm × 5 μm) were scanned and analyzed. All measurements and experiments were at least duplicated over different days with separate cell cultures. Representative examples of α-Syn PFF treatment per concentration are shown in Figure 3. Records of all scans (Figures S3–S6), as well as additional representative bright field images of the SH-SY5Y cells during SICM imaging (Figure S7), are included in the Supporting Information.

**Figure 3:**
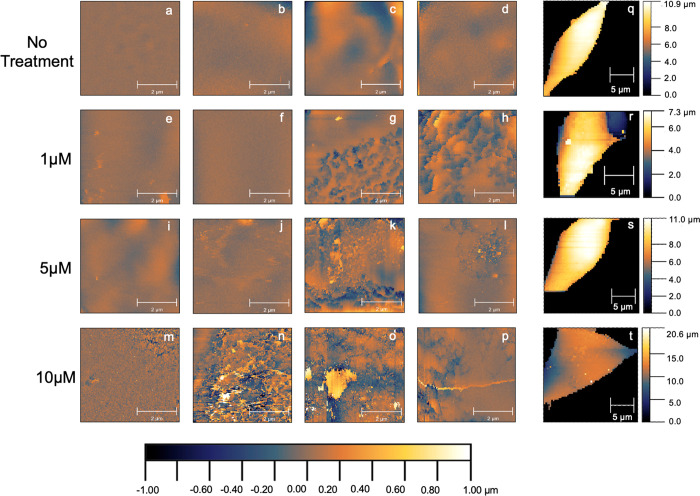
Representative SICM images of SH-SY5Y cell membranes after PFF treatment for 48 h. Images a–p show flattened fine scans of 5 μm × 5 μm sections of the cell membrane, with pixels colored by their deviation from the mean *z*-height. Images q–t show the coarse scans of selected cells. Four experimental conditions are included: no treatment (control) and treatment with three different concentrations of α-Syn PFF (1, 5, and 10 μM). For each condition, one random coarse scan and four random sections from different cells were selected (a–d, q, control; e–h, r, 1 μM; i–l, s, 5 μM; m–p, t, 10 μM).

In the control (no treatment) group, minimal membrane irregularity appears in most sections, consistent with our expectations (Figure 3a–d). These minor changes, in part also observed in the treatment groups, may have resulted from exposure to the fixative (see also Figures S4–S6). In the experimental groups (Figure 3 e-h [1 μM], i–l [5 μM], m–p [10 μM]), membrane roughness visually increases with both negative dips and positive protrusions at the submicrometer level. In the 1 μM treatment sections, dips and protrusions take on a shape reminiscent of membrane folding. In the 5 μM and 10 μM treatment sections, membrane protrusions and larger debris become common and assume a more crystalline appearance, which may indicate the presence of remaining α-Syn PFFs, extracted lipids, or α-Syn PFFs/lipid coaggregates.^14^ These membrane features are somewhat different from disruptions caused by α-Syn oligomers, where large and transient pores were observed at high α-Syn concentrations.^25^

To further quantify the observed differences, membrane roughness was measured from all flattened sections and then statistically analyzed using a bootstrap comparison of mean roughness across all groups (Figure 4A). As expected, most control samples are characterized by low roughness values (1.16–1.6, log/14.45 nm to 39.81 nm), reflective of normal membrane fluctuations and electric current noise. Statistical analyses demonstrate significant differences (*p* < 0.0001) in membrane roughness between control and all α-Syn PFF treated samples. Cells treated with 1 μM PFFs exhibit two populations of data, indicating that some areas of the cell remain relatively uninterrupted while other areas begin to exhibit morphological variations. When treated with higher concentrations (5 μM and 10 μM), all roughness values start to increase, corresponding to the obvious increase in protrusions and large debris present with *p* < 0.0001 for 1 μM/5 μM and 1 μM/10 μM and *p* < 0.05 for 5 μM/10 μM. The latter two concentrations are only 2-fold different, accounting for the larger *p*-value compared to the smaller *p*-value for the concentrations that differed 5- or 10-fold. The conclusion is not impacted by the distribution of imaging areas (Figure S8 and related discussions). We then focused on analyzing the “positive features” (i.e., residues, protrusions, and debris) by extracting the size information on positive features from the cell sections with detectable features using the “grains distributions” function from Gwyddion (Figure S9). The scatter plots of the surface area versus the maximum height (*Z*_max_) of each positive feature under different treatments are shown in Figure 4B,C. There is clearly a concentration-dependent increase in both the number and size of the positive features. These features could be PFF clusters, as seen in AFM characterization (Figure 2A), or lipid-fibril bundles, which have been demonstrated to coaggregate in large masses.^14,16^ To assist in interpreting the SICM observations, we further assessed cell viability.

**Figure 4:**
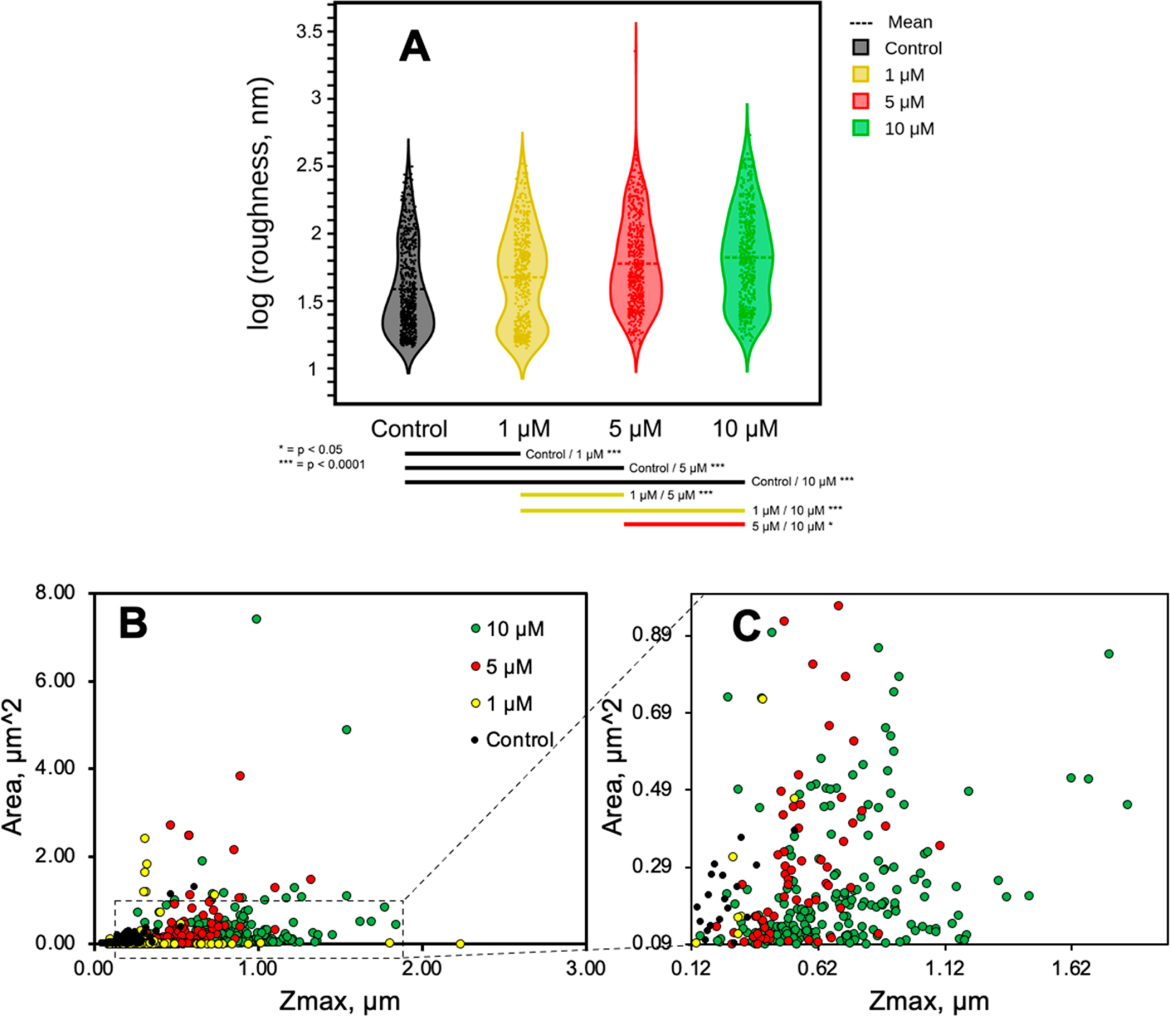
Quantitative analysis of the membrane roughness. (A) Roughness of cell membranes after different PFF treatments. The dark dotted line across each concentration is the mean roughness of each group. Each roughness data point is plotted to its corresponding concentration. The curves represent the frequency of the data points. The statistical significance of the differences between means was assessed via bootstrap sampling. The mean ± 95% confidence interval values are as follows: control = 1.588 ± 0.0264 (*n* = 570); 1 μM = 1.677 ± 0.0354 (*n* = 400); 5 μM = 1.775 ± 0.0291 (*n* = 475); 10 μM = 1.823 ± 0.0356 (*n* = 375). (B, C) Analysis of positive features. (B) Scatter plots of the area versus the maximum height (*Z*_max_) of positive features extracted from cell sections under different treatments and (C) zoom-in of dashed box labeled area in (B). Numbers of features and cell sections analyzed are control, *n* = 138 from 5 sections; 1 μM, *n* = 161 from 6 sections; 5 μM, *n* = 721 from 9 sections; 10 μM, *n* = 2560 from 8 sections.

As presented in Figure 5A, the XTT assay shows only a small though statistically significant decrease in cell viability for all treatments compared to the control group, but there was no statistically significant difference between the three treatments. Similarly, the LDH assay, which quantifies LDH release to cell media upon damage to the plasma membrane as a measure of the cytotoxic effect of the treatment, showed a small increase in LDH release compared to the spontaneous LDH release for all treatments, independent of the PFF concentrations (Figure 5B; an initial pilot experiment can be found in Figure S10). This increase was not statistically significant. For this experimental setup, due to the limitation of the PFF stock solution concentration, a substantial amount of cell culture supernatant was removed during treatment to maintain constant volume, which could have caused cell loss at uncertain levels. The lack of dose dependency might be due to the PFFs clumping together at higher concentrations, preventing them from binding tightly to cell membranes to cause more damage. This also indicates that the increased roughness and amount of positive features observed in SICM images with higher doses are more likely contributed by PFFs clusters than extracted lipids.

**Figure 5:**
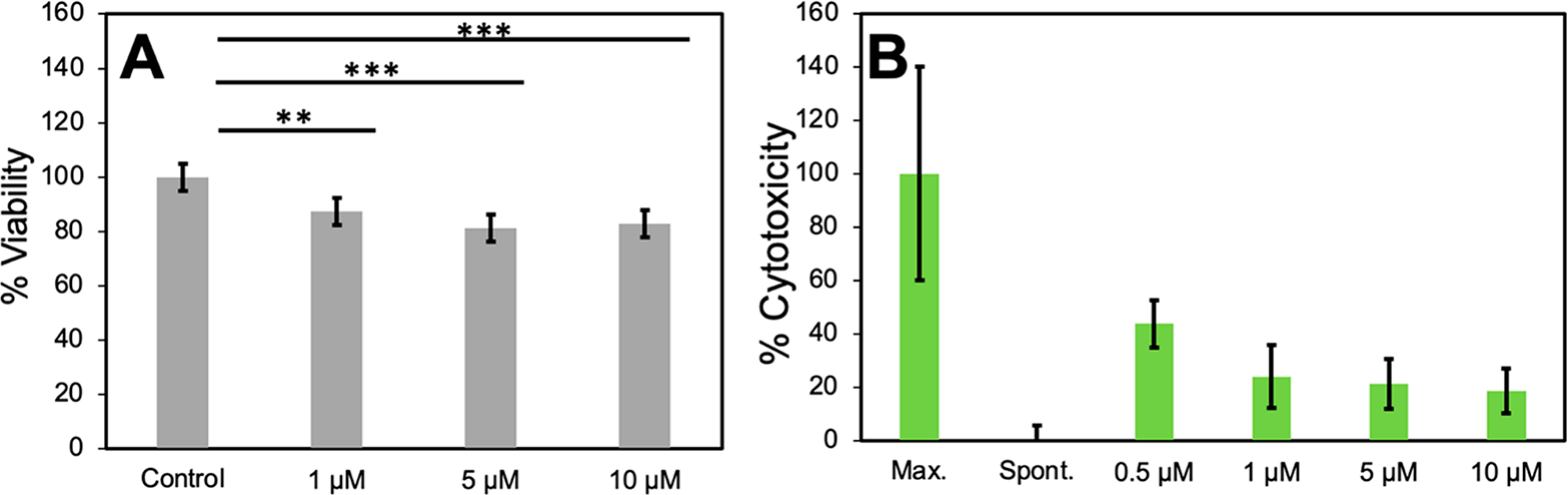
Effects of PFFs exposure on SH-SY5Y cells viability. (A) XTT assay measuring cell viability. (B) LDH release assay measuring cytotoxicity. Data are the mean ± SD, *n* = 3: **p < 0.01 and ***p < 0.001.

Our study was limited to imaging the end point roughness change after 48 h because the cells could not survive for a prolonged time in the SICM setup without incubation conditions. In future work, we plan to solve these limitations by integrating a live cell imaging chamber, which will mimic incubation environments by regulating the temperature, CO_2_ conditions, and pH of the imaging buffer solution. This will enable us to carry out long-term monitoring of live cell dynamics and to fully exclude the effect of fixative agents. Furthermore, our current study only covers the initial stage of PFFs accumulating at the cell membranes, while the prion-like spreading mechanism of PD suggests that exogenous PFFs recruit endogenous α-synuclein into pathological aggregates.^29^ Therefore, further inquiries into possible endogenous α-Syn coactors should be made, and molecular-level characterization with surface plasmon resonance microscopy and fluorescence microscopy could be employed to provide more comprehensive evidence.

To summarize, this work has provided insight into the effects of α-Syn PFFs exposure on SH-SY5Y cell membranes. A significant membrane roughness increase occurred for all treatment groups after exposure to α-Syn PFFs for 48 h compared to control groups. As concentration increased, roughness also increased with an abundance of crystalline debris and/or large protrusions. XTT and LDH assays showed that this was not accompanied by pronounced cytotoxic effects, suggesting that PFFs may affect cell membranes and disrupt cell function without causing prominent cell death. The lack of dose-dependent cytotoxic effects suggests that the increased roughness and positive features observed in SICM images with higher doses are likely contributed by PFFs accumulating on the cell membranes, which may have led to membrane curvature changes rather than direct disruption of the lipid bilayer. These conclusions also reinforce previous works that have shown that α-Syn fibrils tend to remain on the surface of cell membranes and have weaker binding affinity to lipid bilayers compared to cytotoxic α-Syn oligomers.^3,15^ Our observations with PFFs at SY-SY5Y cells are consistent with reported work that injection of PFFs does not lead to substantial neuron death within 48 h at a primary neuronal model.^29^ This suggests possible treatment windows in early Parkinson’s disease that may target α-Syn before the neurons are lost, such as using L-DOPA or other dopamine agonists to stabilize α-Syn fibrils to prevent the accumulation and further aggregation of pathological α-Syn.^30,31^

## Methods

### Sample Preparation

SH-SY5Y neuroblastoma cells were purchased from ATCC (CRL-2266, Manassas, VA). Cells were grown under standard culture conditions (37 °C and 5% carbon dioxide) in 89% v/v Dulbecco’s modified Eagle medium with Ham’s F-12, 10% v/v fetal bovine serum, and 1% v/v 1× penicillin–streptomycin solution and differentiated with all-trans retinoic acid (10 μM) (Thermo Scientific, Waltham, MA). Cells were then cultured on cell culture-treated dishes (35 mm, Thermo Scientific, Waltham, MA) with a silicon piece containing four wells (Sarstedt, Germany) to isolate samples into four areas and enable the treatment of different concentrations of PFFs under the same culturing conditions, as shown in Figure 1B.

α-Syn PFFs were purchased from StressMarq Biosciences Inc. (Canada, type 2, SPR-317), thawed and sonicated, and dissolved in cell culture media as a stock solution (35.7 μM). For AFM characterization of the length distribution, PFFs from the stock solution were deposited on a mica sheet and imaged in noncontact air mode with a Park NX12 multifunctional microscopy platform equipped with a detachable AFM head (Park Systems, Seoul, South Korea). The acquired images were linearly flattened with Gwyddion version 2.51 (http://gwyddion.net/).

Varying concentrations of α-Syn PFFs were applied to the cells, which were then incubated for approximately 48 h prior to SICM imaging or cell viability assessment. Prior to imaging, cells were fixed with 1% paraformaldehyde for 30 min, rinsed three times with filtered phosphate buffer saline (PBS), then fully submerged in filtered PBS.

### SICM

Nanopipette probes were pulled from quartz capillaries to give tip radii of approximately 70–100 nm (Figure S1), filled with filtered PBS solution, and fitted with an Ag/AgCl electrode. All images were acquired with a Park NX12 multifunctional microscopy platform equipped with a detachable SICM head, mounted on a Nikon Ti-U inverted optical microscope (Nikon Inc.), and operated with SmartScan (Park Systems). Images were acquired in approach–retract–scan (ARS) mode with a threshold of 1% current change.

Fine scans were flattened according to a two-dimensional polynomial profile using Gwyddion version 2.59 (http://gwyddion.net/).^25^ Roughness was measured across the membrane surface in 1 μm × 1 μm sections as the root-mean-square deviation of *z*-heights from the average. To compare how roughness differs between groups, a mean difference bootstrapping analysis was done in R version 4.1.1 (R Core Team, Vienna, Austria) with 10 000 bootstrap samples per group. “Positive feature” analysis was done through Gwyddion with the “grains distributions” function.

### XTT and LDH Assays

XTT assay (Invitrogen CyQUANT XTT Cell Viability Assay, Fisher Scientific) and LDH assay (CyQUANT LDH Cytotoxicity Assay, Fisher Scientific) were performed on SH-SY5Y neuroblastoma cells following the vendor’s instructions. All data were expressed as mean ± standard deviation. Comparisons between the different groups were performed by One-Way ANOVA followed by Bonferroni’s post comparison test by using IBM SPSS Statistics software version 27.

Detailed information regarding the methods can be found in the Supporting Information.

## Supporting Information Available

Additional details for methods and supplementary data (PDF)

## Abbreviations

α-Syn: α-synuclein
PD: Parkinson’s disease
PBS: phosphate buffered saline
SICM: scanning ion conductance microscopy
XTT: sodium (2,3-bis(2-methoxy-4-nitro-5-sulfophenyl)-2*H*-tetrazolium-5-carboxanilide)
LDH: lactate dehydrogenase
