# Supplementary material for: Observation of α-Synuclein Preformed Fibrils Interacting with SH-SY5Y Neuroblastoma Cell Membranes Using Scanning Ion Conductance Microscopy
Source: ACS Chem Neurosci. 2022 Dec 1;13(24):3547–53. doi: 10.1021/acschemneuro.2c00478 (PMC9782390; doi:10.1021/acschemneuro.2c00478)
Supplement: Supplementary file 1 — cn2c00478_si_001.pdf [file cn2c00478_si_001.pdf]

Supporting Information

Observation of Alpha-Synuclein Pre-Formed Fibrils Interacting with SH-SY5Y Neuroblastoma Cells Membranes Using Scanning Ion Conductance microscopy

Christina Feng[1], Marisol Flores[1], Christina Dhoj[1], Adaly Garcia[1], Sheehan Belleca[1], Dana Abou Abbas[2] Jacob Parres-Gold[1a], Aimee Anguiano[1], Edith Porter[2], and Yixian Wang[1]*

[1] Department of Chemistry and Biochemistry, California State University, Los Angeles, Los Angeles, California 90032, United States
[2] Department of Biological Sciences, California State University, Los Angeles, Los Angeles, California 90032, United States

*Corresponding Author: ywang184@calstatela.edu

METHODS

Chemicals and Materials

Ethanol (anhydrous), 0.25% trypsin, Dulbecco's Modified Eagle Medium with Ham's F-12 (DMEM/F-12 50/50 mix, L-glutamine, HEPES supplemented) medium, fetal bovine serum (FBS), all-trans retinoic acid, nickel chloride, penicillin-streptomycin (Gibco, 10,000 units/mL penicillin and 10 mg/mL streptomycin) and phosphate buffer saline (PBS) tablets were purchased from Thermo Fisher Scientific (Waltham, MA). Cell culturing medium was made with 89% v/v DMEM/F12 medium, 10% v/v FBS, and 1% v/v penicillin-streptomycin. All solutions were made using double-deionized water (resistivity = 18.2 MΩ*cm at 25 °C, Milli-Q Ultrapure water EQ 7000 Purification System, MilliporeSigma, Burlington, MA). PBS solutions prepared from tablets were filtered through a 0.02 µm pore size syringe filter prior to imaging (Thermo Fisher Scientific, Waltham, MA).

α-Syn PFFs and AFM Characterization

Type 2 alpha-synuclein pre-formed fibrils (catalog no. SPR-317) were purchased from StressMarq Biosciences Incorporated (British Columbia, CAN). Frozen PFFs were sonicated in a 37°C water bath for a 30 sec on - 30 sec off - 30 sec on - 30 sec off - 1 min on procedure to briefly thaw and release fibrils. Two hundred µL of cell culturing medium was added directly to the vial in sterile conditions, creating a 35.7 µM stock solution, which was stored at - 20°C until needed.

The length distribution of the α-Syn PFFs in the stock solution was analyzed by AFM. A muscovite mica substrate (V-1 quality, Electron Microscopy Sciences, Hatfield, PA) was cleaved with scotch tape and rinsed thoroughly with DI water. The sheet was dried with nitrogen gas and incubated with nickel chloride (10 mM) for 20 minutes before drying under a stream of nitrogen gas. The substrate was incubated with 20 µL of PFF stock solution for 20 minutes, gently rinsed with DI water, and dried under a stream of nitrogen gas. Fibrils were imaged in non-contact air mode with a Park NX12 multifunctional microscopy platform (Park Systems, Seoul, South Korea) equipped with an AFM head attachment and mounted on a Nikon Ti-U inverted optical microscope (Nikon Inc., Tokyo, Japan). Images were acquired using a PPP-NCHR non-contact cantilever (force constant 42 N/m, Park Systems) at 256 × 256-pixel resolution with a scan rate of 0.5 Hz and a set point of approximately 10.2 nm using SmartScan (Park Systems). The acquired images were processed through XEI version 1.8.2 (Park Systems). Fibril lengths were measured using the "Grains" function in XEI with a minimum threshold of 0.5 nm in height. The resulting length histograms were acquired using Microsoft Excel.

SH-SY5Y Neuroblastoma Cell Culture and α-Syn Exposure

SH-SY5Y neuroblastoma cells were purchased from American Type Culture Collection (ATCC, Manassas, VA). Cells were grown under standard culture conditions (37 °C and 5% carbon dioxide) in cell culture media. Trypsin (Corning® 0.25% trypsin, 2.21 mM EDTA, 1X) was used to detach cells from culture plates for sample splitting and passaging. Before fibril treatment, cell differentiation was induced using a protocol adapted from Kovalevich & Langford.[S1] Cell media was supplemented with all-trans retinoic acid (10 μM) and incubated with plated cells for 48 hours until visible neuronal projections were seen. For SICM imaging, SH-SY5Y cells were cultured on ThermoScientific BioLite Cell Culture Treated Dishes (Waltham MA), which have a polyethylene substrate with cell culture surface treatment to enhance cell adhesion. A silicon piece containing four wells (cut from flexiPERM slides micro 12, purchased from Sarstedt, Germany) was loaded on the culturing dish to isolate samples into four areas and enable the treatment of different concentrations of PFFs under the same culturing conditions, as shown in Figure 1B. For PFF treatment, varying concentrations of α-Syn PFFs were applied from the stock solution to the cells, which were then incubated for approximately 48 hours prior to SICM imaging.

Nanopipette Probes for Scanning Ion Conductance Microscopy

Nanopipette probes were pulled from quartz capillaries to give tip radii of approximately 70−100 nm. SICM nanopipette probes (70-100 nm in radius) were pulled from quartz capillaries (1.0 mm outer diameter, 0.50 mm inner diameter, 7.5 cm length, Sutter Instruments, Novato, CA) using a P-2000 laser-based micropipette puller (Sutter Instruments). Typical pulling parameters are as follows: heat = 780, filament = 4, velocity = 15, delay = 120, pull = 115. Nanopipettes were filled with filtered PBS solution and fitted with an Ag/AgCl electrode.

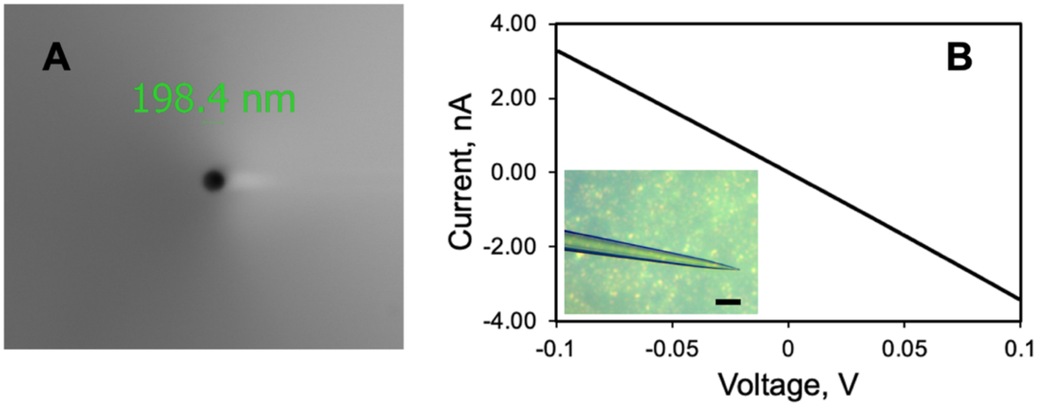

Figure S1. Nanopipette characterization with (A) SEM and (B) current-voltage curve. SEM images were taken by the Boettcher group from University of Oregon using an environmental SEM (ThermoFisher Apreo 2S, Waltham, MA) at ~2.82e-2 Pa. Current-voltage curves were obtained via a potentiostat (CHI610E; CH Instruments, Austin, TX, United States) by filling the nanopipettes with 0.1 M KCl and immersing them to the same solution and applying linear sweep between the two Ag/AgCl electrodes inserted into the pipette and the outside solution, respectively. Inset in B is a 10X optical image of the nanopipette captured with a digital microscope (OMAX - MD82ES10), scale bar = 20 μm.

Figure S1A shows a representative SEM of a pipette with a diameter of 198.4 nm (radius 99.2 nm). Nanopipette size can also be estimated based on a current-voltage curve, as shown in Figure S1B. The resistance extracted from the current-voltage curve is related to the nanopipette radius based on the following equation[S2]

$$R = \frac{1}{4\kappa a} + \frac{1}{\pi \kappa a tan\theta} \qquad (S1)$$

where R is the total resistance that can be determined from the current-voltage curve, κ the solution conductivity, which is 1.29 Sm^{-1} for 0.1 M KCl, a the radius of the pipette, and θ the pipette angle at the tip. By assuming $\tan\theta$ to be 0.1 based on optical observation and the resistance extracted from the current-voltage curve, 29.8 MΩ, the pipette radius is estimated to be 89 nm, which is similar to the SEM result, demonstrating the ionic current can provide a good estimation of the nanopipette size.

Pipettes of this size usually show around 3 nA ionic current at a 0.1V bias in PBS solution during SICM scans. We typically use nanopipettes that give an ionic current of 2-3 nA under the same condition. The estimated size range would be 70 – 100 nm in radius for all the SICM tests. Therefore, the lateral resolution of the SICM images is < 300nm[S3]. We determined the axial resolution to be around 120 nm based on the peak-to-peak noise level of 40 nm. As a result, in Figure 4C, features smaller than 0.09 μm^2 in area and 120 nm in height were excluded.

Scanning Ion Conductance Microscopy of Neuroblastoma Cells

Prior to imaging, cells were fixed with 1% paraformaldehyde dissolved in PBS for 30 minutes, rinsed three times with filtered PBS, then fully submerged in filtered PBS. Images were gathered immediately afterward. For each condition, an image covering a major portion of a cell (coarse scan, typically 10 to 20 μm in dimension, 64 × 64 pixel by pixel) was taken first for most of the samples, then multiple 5 μm × 5 μm cell sections were scanned (fine scans, 128 × 128 pixel by pixel) to observe any protrusions/disruption within the cell at a better resolution (~40 nm pixel resolution).

All images were acquired with a Park NX12 multifunctional microscopy platform (Park Systems) equipped with a detachable SICM head, mounted on a Nikon Ti-U inverted optical microscope (Nikon Inc.), and operated with SmartScan (Park Systems). A CCD camera (Pike F-032B, Allied Vision, Exton, PA) was connected to the optical microscope to assist in probe positioning. The nanopipette was immersed in the PBS solution, and a potential bias was applied between the electrode inside the nanopipette and the second Ag/AgCl electrode in the PBS solution to reach an ionic current of 1 nA. After the nanopipette was positioned above a cell, it was approached incrementally in approach-retract-scan (ARS) mode with a set point of 1%. ARS mode enabled the probe would incrementally approach the cell to prevent crashing. Coarse scans were typically completed within 8-10 minutes. Fine scans were typically completed within 15-20 minutes. It would typically take 4 hours to finish scanning multiple areas within each sample. All experimental groups contain cell areas that were imaged from the full range of times to eliminate the potential for time bias. Cells were assessed microscopically at the beginning and the end of the analysis, and cells that had rounded up were not included in the analysis.

XTT assay

XTT assay (Invitrogen™ CyQUANT™ XTT Cell Viability Assay, Fisher Scientific) was performed on SH-SY5Y neuroblastoma cells according to the manufacturer's instructions. Briefly, the cells were grown in 96-well, flat bottom tissue culture treated plates (Corning™ Costar™) in DMEM/F-12 50/50 mix, supplemented with 10% fetal bovine serum and 1% penicillin/streptomycin. The cells were incubated at 37°C at 5% CO_2 for 24 hours before adding the fibril treatment. The PFFs were sonicated as mentioned above, and growth media was added to the vial before adding fibrils into the wells at final concentrations of 1, 5, and 10 μM and incubated for 48 hours. After 48 hours, the XTT solution was added and incubated for 4 hours before reading with the Victor X3 multiplate reader (PerkinElmer, Waltham, MA) at 450 nm. Data were extracted and plotted using Microsoft Excel. Comparisons between the different groups were performed by One-Way ANOVA followed by Bonferroni's post-comparison test using IBM SPSS Statistics software version 27.

LDH Assay

Cell cytotoxicity of SH-SY5Y cells upon PFFs treatment was measured using CyQUANT™ LDH Cytotoxicity Assay from ThermoFisher Scientific (Hanover Park, IL) according to the manufacturer's instructions. Briefly, the cells were plated in a 96-well flat bottom tissue culture treated plate (Corning™ Costar™) at a density of 1.67 x 10^4 cells/well. After 24-hour incubation at 37ºC and 5.0% CO_2, sets of triplicates were treated with PFFs with various concentrations to achieve 0.5, 1, 5, and 10 μM final concentration. Another set of triplicates, which measure spontaneous LDH release (negative control), was treated with sterile water to account for the spontaneous lysis. After the 48-hour treatment incubation, the last set of triplicates was treated with 10X Lysis buffer to measure maximum LDH release and incubated for additional 45 minutes. Afterward, 50 μL media was removed from each well and transferred to a new 96-well plate. The media was mixed with a 50 μL LDH reaction mixture, incubated further for 30 minutes at room temperature, and protected from light. The absorbance was monitored using the VictorX spectrophotometer at 490 nm within 30 minutes after adding 50 μL of the stop solution. Cytotoxicity with each treatment was calculated using the following equation:

$$\%\, Cytotoxicity = \frac{A_{treat} - A_{spon,mean}}{A_{max,mean} - A_{spon,mean}} \times 100 \qquad \text{(S2)}$$

where A_{treat}, $A_{spon,mean}$, and $A_{max,mean}$ represent the absorbance from each treatment, mean of all spontaneous LDH release, and mean of all maximum LDH release, respectively. Comparisons between the different groups were performed by One-Way ANOVA followed by Bonferroni's post-comparison test using IBM SPSS Statistics software Version 27.

SUPPLEMENTARY DATA

Vendor provided characterization information of PFFs

The following figure (https://www.stressmarq.com/products/protein/alpha-synuclein-protein-spr-317/?v=7516fd43adaa) shows the vendor-provided circular dichroism (CD) spectra, which indicates significant β-sheet content (43.0%) of Type 2 PFFs that we used in this study.

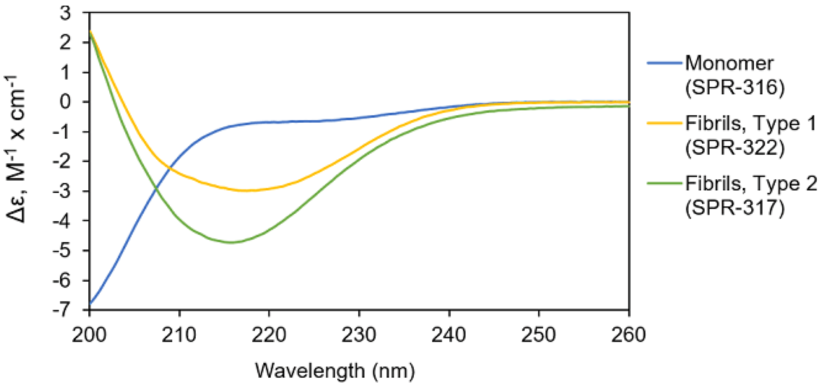

Secondary structure as analyzed by CDNN deconvolution of 200-260nm spectral range

	Fibrils, Type 1 (SPR-322)	Fibrils, Type 2 (SPR-317)
Alpha helix	23.7%	19.6%
Beta sheet/turn	40.4%	43.0%
Random Coil	31.4%	33.4%
Total Sum	95.5%	96.0%

Figure S2. Vendor-provided CD characterization of PFFs. Used with permission from StressMarq Biosciences Inc. © 2022 www.stressmarq.com.

A full record of all scanning ion conductance microscopy (SICM) scans

Figures S3-6 show all the SICM scans from the control (3 trials, 8 cells, 23 fine scans), 1 µM treatment (2 trials, 6 cells, 16 fine scans), 5 µM treatment (2 trials, 9 cells, 19 fine scans), and 10 µM treatment (2 trials, 6 cells, 15 fine scans). Please note that all 5 µm × 5 µm fine scans were flattened according to a two-dimensional polynomial profile and aligned with Gwyddion version 2.51, setting the average z-height to 0 µm. This process accounts for the overall curvature of the cell membrane by fitting the z-height data to polynomials in both the x and y directions and has been demonstrated capable of preserving local features on the membrane while eliminating overall membrane curvature in our previous work.[S4] Some of the fine scans in Fig. S3 were taken directly without a coarse scan due to time constraints.

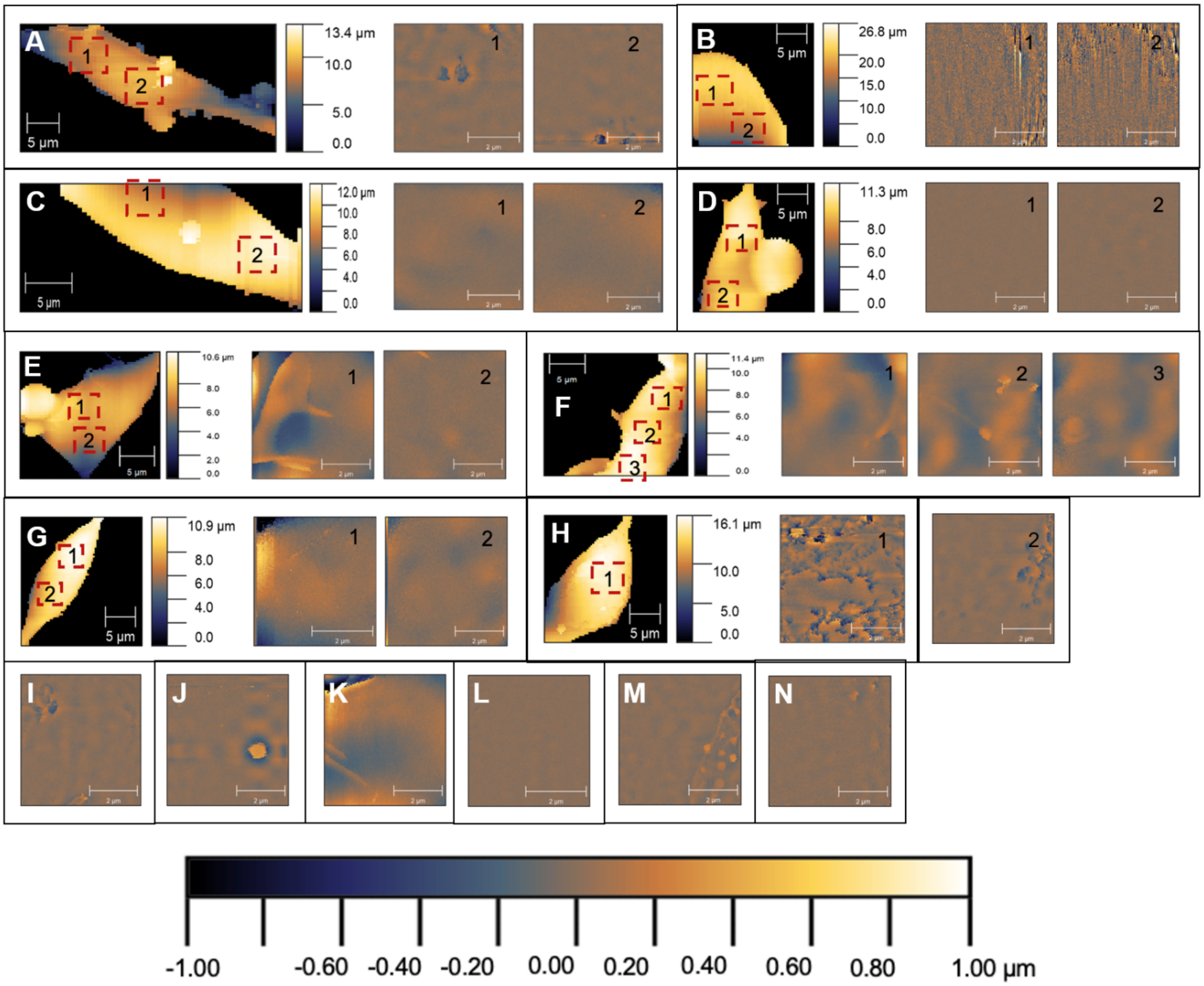

Figure S3. A full record of SICM images of SH-SY5Y cells without PFFs treatment. Each panel with a black border line includes a coarse scan (64 × 64 pixel by pixel) of a major portion of a cell and several fine scans (128 × 128 pixel by pixel, 5 µm × 5 µm) of the sections as labeled. All fine scans were flattened through Gwyddion.

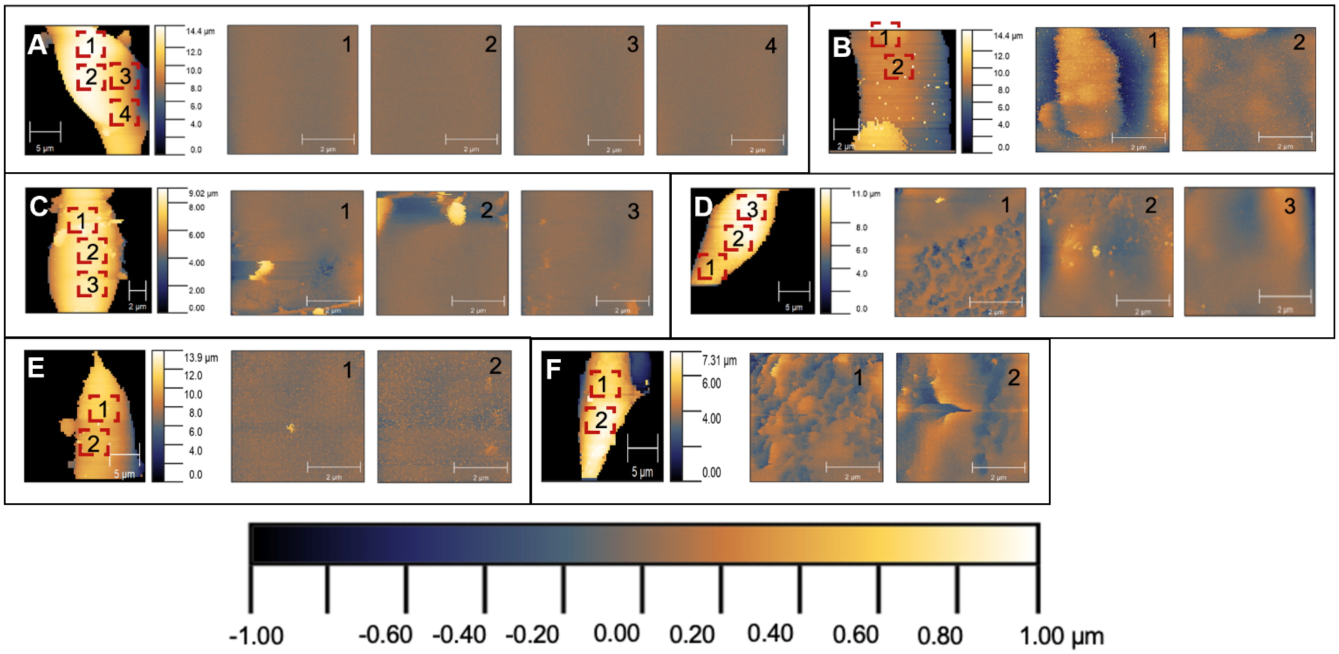

Figure S4. A full record of SICM images of SH-SY5Y cells with 1μM PFFs treatment. Each panel with a black border line includes a coarse scan (64 × 64 pixel by pixel) of a major portion of a cell and several fine scans (128 × 128 pixel by pixel, 5 μm × 5 μm) of the sections as labeled. All fine scans were flattened through Gwyddion.

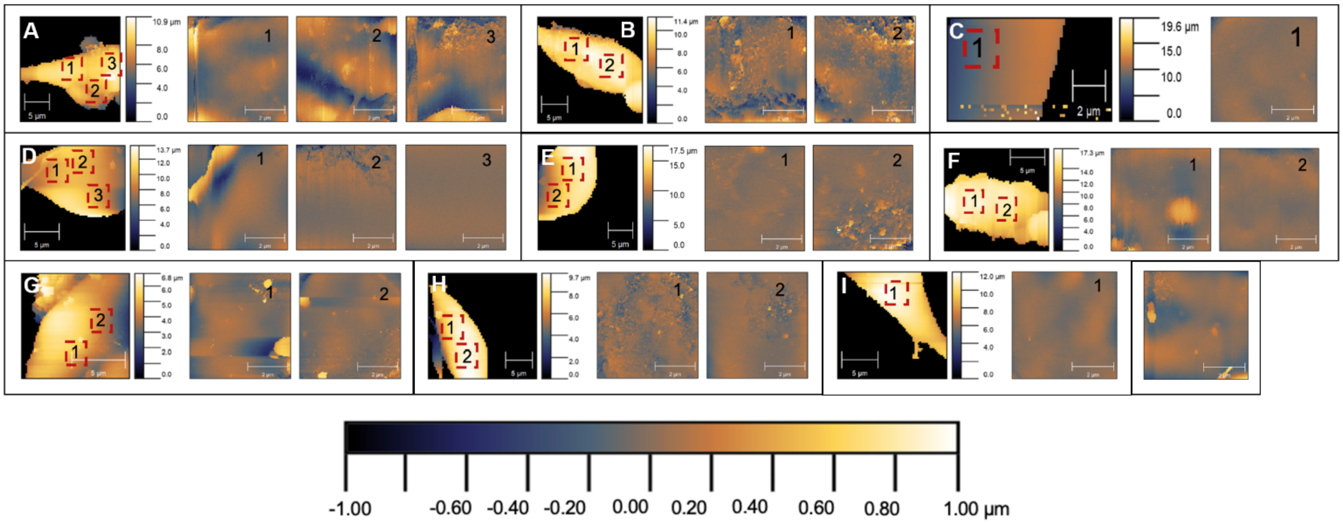

Figure S5. A full record of SICM images of SH-SY5Y cells with 5μM PFFs treatment. Each panel with a black border line includes a coarse scan (64 × 64 pixel by pixel) of a major portion of a cell and several fine scans (128 × 128 pixel by pixel, 5 μm × 5 μm) of the sections as labeled. All fine scans were flattened through Gwyddion.

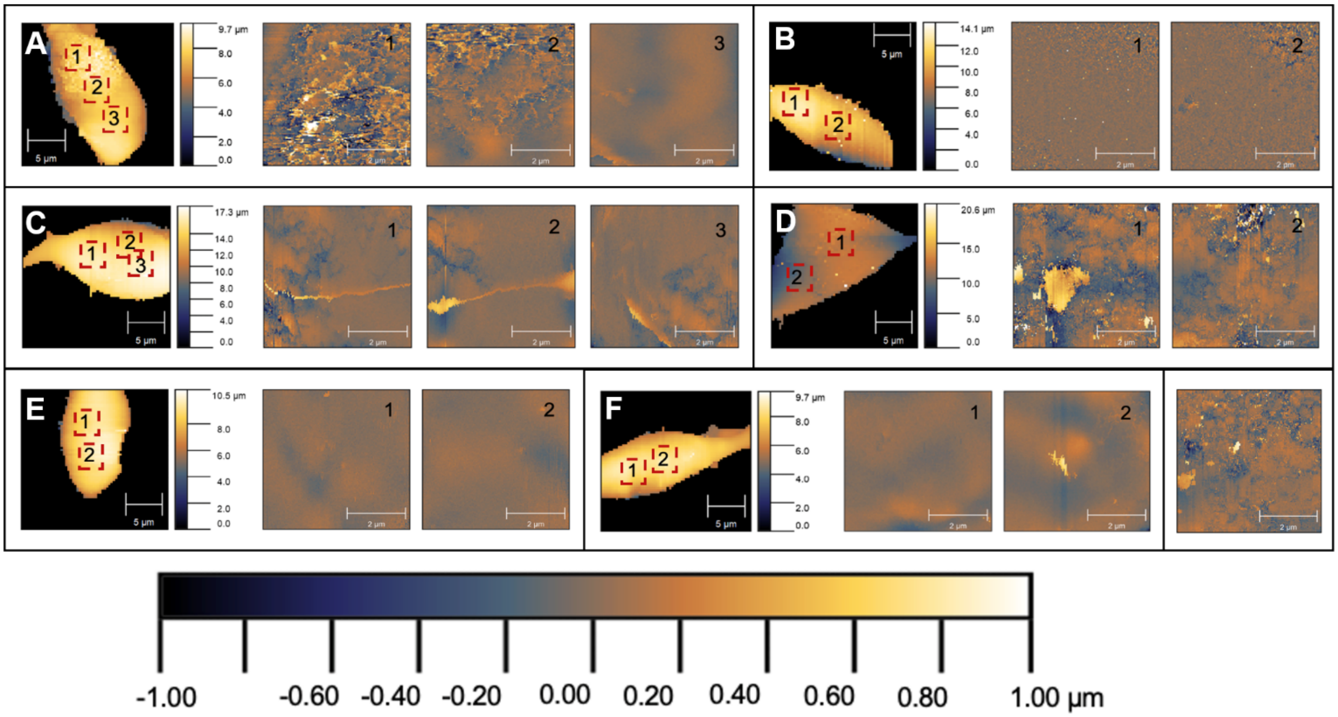

Figure S6. A full record of SICM images of SH-SY5Y cells with 10µM PFFs treatment. Each panel with a black border line includes a coarse scan (64 × 64 pixel by pixel) of a major portion of a cell and several fine scans (128 × 128 pixel by pixel, 5 × 5 µm) of the sections as labeled. All fine scans were flattened through Gwyddion.

Representative brightfield images of SH-SY5Y cells during SICM imaging

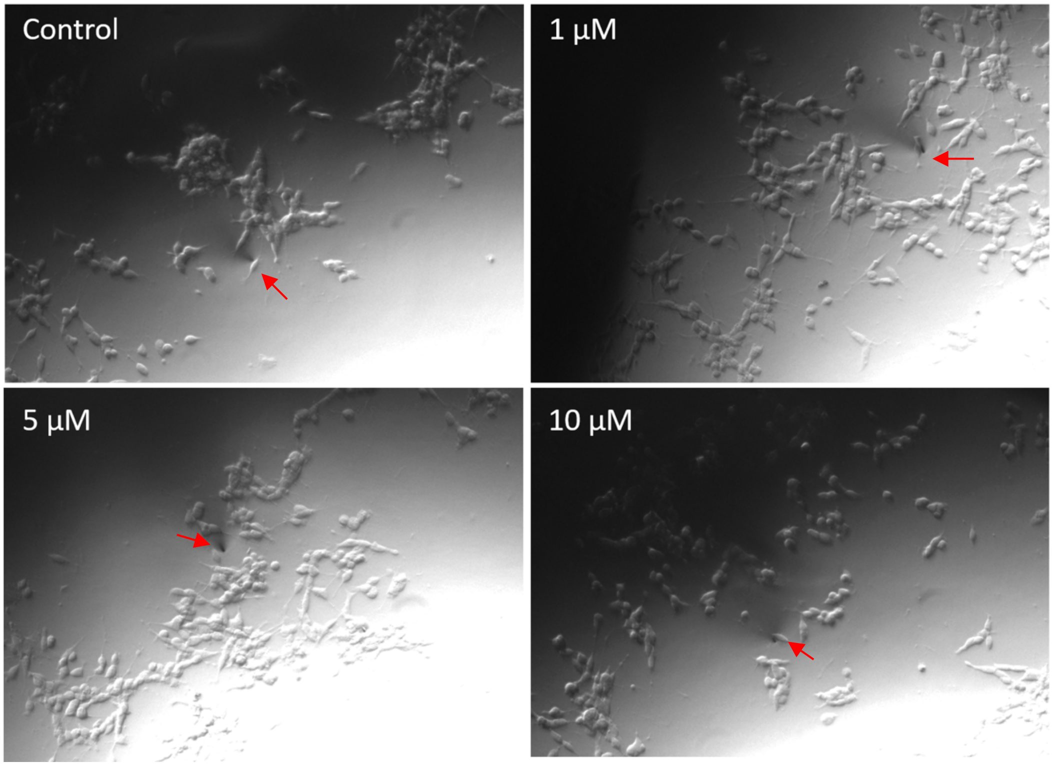

Figure S7. Representative brightfield images of SH-SY5Y cells post-fixation during SICM imaging. Cell coverage varied between experiments, but scans aimed to image cells relatively isolated to prevent probe crashing and interference, as shown with red arrows.

Comparison of center and edge areas

We analyzed the roughness differences between center and edge areas in all groups; please note that "edge" and "center" areas are hard to classify/define within images. Therefore, the following analysis is, to a certain degree, subjective. In general, areas that were close to the synapse of the cell were classified as edge, while areas closer to the cell body were classified as center, as shown in Figure S8.

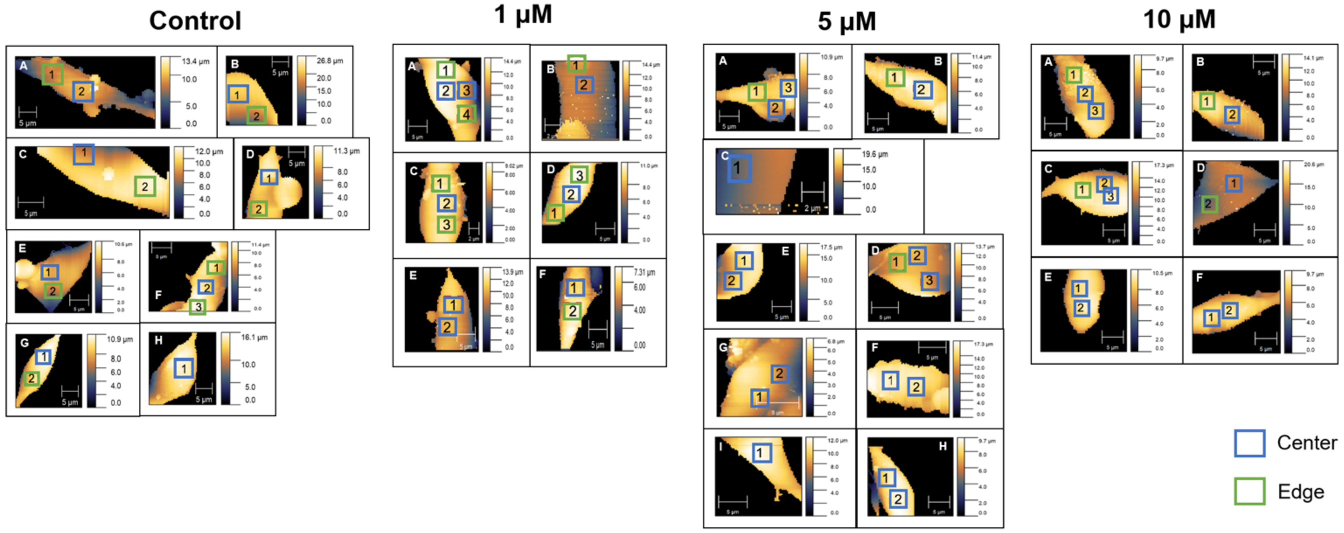

Figure S8. Identification of center and edge areas from the SICM whole cell images (64 × 64 pixels) of each condition adapted from Figures S3-S6. Areas are subjectively labeled; areas visually determined to be nearer to the cell body ("center") are labeled in blue, and areas nearer to synapses ("edge") are labeled in green. Two-tailed t-tests were conducted to determine if each group's "center" and "edge" areas have significantly different roughness values.

Table S1. Comparison of the roughness from center and edge areas for different treatment groups

	Center (log of mean)	Edge (log of mean)	Two-tailed p-value
Control	1.65 (n=200)	1.66 (n=200)	0.69
1 μM	1.67 (n=200)	1.68 (n=200)	0.79
5 μM	1.74 (n=375)	1.97 (n=75)	1.86×10^{-7}
10 μM	1.72 (n=250)	1.92 (n=100)	1.39×10^{-6}

As shown in Table S1, there are no statistical differences between center and edge areas for control and 1 μM groups, while there were statistical differences between center and edge areas for 5 μM and 10 μM groups. However, this should not affect our conclusion of dose-dependent roughness increase because (1) approximately three times more center areas were taken for 5 μM and 10 μM groups, and these center areas exhibited lower average roughness than edge groups. Therefore, if we took similar numbers of edge areas and center areas like the control and 1 μM groups, the average roughness for the 5 or 10 μM groups would increase; this would make the differences between control and α-Syn PFF-treated groups even more statistically significant, which would reinforce our conclusion; (2) the bootstrap analysis has taken into account the possible heterogeneity of roughness values in cells when calculating statistical significance and assumed that cell membrane values are non-normally distributed.

Additional information for "positive feature" analysis

"Positive feature" analysis aims to extract size information from all protrusions and debris. This was done through Gwyddion with the "Grains Distributions" function. Features were selected from flattened images and masked manually based on height. As shown in Fig. S9, the red areas are masked protrusions, from which the surface area and maximum height were analyzed.

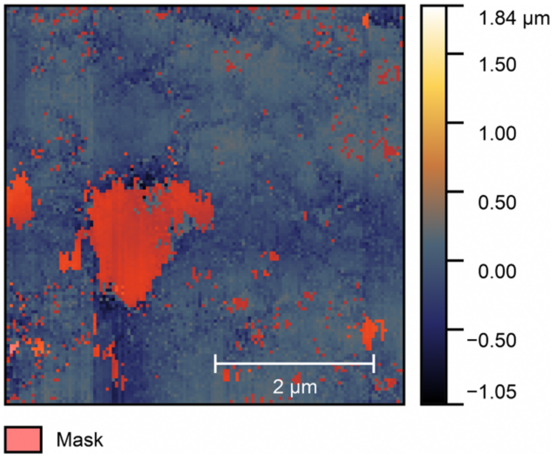

Figure S9. Representative masking process of a fine scan from a 10 µM treatment. Surface area and maximum height were analyzed for each separate red mask within an image.

Initial pilot experiment for LDH assay

In this pilot experiment, the final PFF concentrations were 1 µM, 4.4 µM, and 7.8 µM. The observation was the same as in Figure 5B.

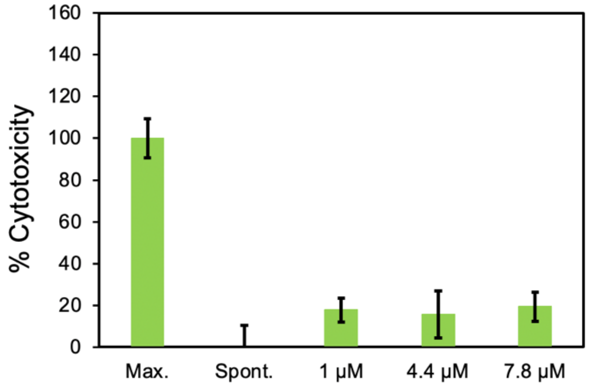

Figure S10. Initial pilot LDH release assay. Data are means ± SD, n =3.

References

(S1) Kovalevich, J.; Langford, D. Considerations for the use of sh-sy5y neuroblastoma cells in neurobiology; 2013; Vol. 1078, pp 9–21.

(S2) Wang, Y.; Wang, D.; Mirkin, M. V. Resistive-pulse and rectification sensing with glass and carbon nanopipettes. *Proc. R. Soc. A Math. Phys. Eng. Sci.* **2017**, *473* (2199), 20160931.

(S3) Rheinlaender, J.; Schäffer, T. E. Lateral resolution and image formation in scanning ion conductance

microscopy. *Anal. Chem.* **2015**, *87* (14), 7117–7124.

(S4) Parres-Gold, J.; Chieng, A.; Wong Su, S.; Wang, Y. Real-time characterization of cell membrane disruption by α-synuclein oligomers in live sh-sy5y neuroblastoma cells. *ACS Chem. Neurosci.* **2020**, *11* (17), 2528–2534.
